# Full-length gene polymorphism of the non-classical HLA-E in Estonian individuals

**DOI:** 10.1007/s00251-025-01381-z

**Published:** 2025-07-10

**Authors:** Timo I. Olieslagers, Ingrid Tagen, Mathijs Groeneweg, Marcel G. J. Tilanus, Lotte Wieten, Christina E. M. Voorter

**Affiliations:** 1 Maastricht University Medical Center+, Transplantation Immunology, Tissue Typing Laboratory, Maastricht, the Netherlands; 2 GROW - Research Institute for Oncology and Reproduction, Maastricht University Medical Center+, Maastricht, the Netherlands; 3 United Laboratories, Tartu University Hospitals, Tartu, Estonia

**Keywords:** HLA-E, Estonia, Allele frequency, Population diversity, Sequencing

## Abstract

Estonia is a small country in the Baltic region of Northern Europe with 1.3 million inhabitants. As a coastal area, the population of Estonia was subjected to migration influences. Due to this admixture of populations, HLA gene diversity in Estonia is interesting to study with regard to allele frequencies, haplotypes, and polymorphism. In this study, we focused on *HLA-E* polymorphism within the Estonian population and compared these with the polymorphism identified in other populations. Full-length *HLA-E* sequencing of 143 individuals originating from Estonia show dimorphism frequencies at amino acid position 107 (0.55 R vs 0.45 G) comparable to other populations. Within the study population, 16 different *HLA-E* alleles were identified, including four novel alleles. These 16 alleles encode four different protein variants. Despite a strong differentiation between the South-East and the rest of the Estonian country, no allele frequency differences for *HLA-E* between these regions were identified. Comparing the allele and SNP frequencies to frequencies found in the different neighboring countries revealed no major differences, except for the SNP encoding for HLA-E*01:06. Association analysis between classical HLA class I genes and polymorphism at amino acid position 107 of *HLA-E* revealed higher frequencies of HLA-A*01 with R107 and HLA-A*03 and HLA-C*04 with G107. In summary, our study provides new insights into *HLA-E* variation within the Estonian population and demonstrates that its level of polymorphism is comparable to those observed in other global populations.

## Introduction

Human Leucocyte Antigen-E (HLA-E) belongs to the group of non-classical MHC class I molecules (class Ib) that play an essential role in immunomodulation. The expression of HLA-E shows a wide tissue distribution; healthy nucleated cells positive for class I also express HLA-E, albeit to a lower level than the classical class I molecules (Wieten et al. 2014). The structure of the HLA-E molecule resembles that of classical HLA class I molecules with three extracellular domains pairing with the β2 microglobulin (O’Callaghan et al. 1998; Koller et al. 1988). HLA-E primarily binds self-peptides derived from the leader sequences of classical class I molecules, thereby monitoring their expression (Sullivan et al. 2006; O’Callaghan et al. 1998). HLA-E has a dual function, playing a role in both the innate and adaptive immune response (Sullivan et al. 2008; van Hall et al. 2010; Wieten et al. 2014; Kraemer et al. 2014). On the one hand, it serves as a ligand for both activating and inhibitory CD94/NKG2 receptors present on NK cells and on a subset of T cells. On the other hand, it interacts with both cytotoxic and regulatory CD8 T cells via their αβ T cell receptor (Wieten et al. 2014; Joosten et al. 2016; Grant et al. 2020).

The *HLA-E* gene is located on the short arm of chromosome 6 between the *HLA-A* and *–C* genes. The HLA genes are among the most polymorphic genes in the human genome, but *HLA-E* is more conserved than classical HLA class I genes and has only a limited level of allelic variation. At present, 378 different *HLA-E* alleles have been assigned, but due to multiple synonymous substitutions and a non-expressed (null) allele, they encode only 142 protein variants (IPD-IMGT/HLA Database v3.60) (Barker et al. 2023). For a long time, only two HLA-E protein variants were known that differ by a single amino acid (Arg/Gly) at codon 107 of the α2 domain: HLA-E*01:01 and *01:03. This is attributed to the predominant focus of studies on sequencing exons 2 and 3 of the gene, which constitute the region encoding the peptide-binding groove of the protein. The rapid development of the sequencing technology and the availability of samples from different populations resulted in the discovery of a more extended variability and the identification of more HLA-E protein variants, including the variants that we and others identified in previous studies examining the full-length *HLA-E* gene polymorphism in individuals from different populations (Olieslagers et al. 2017; Castelli et al. 2015; Felicio et al. 2014; Lucas et al. 2020). Recently, two large studies have confirmed this higher variability: Sauter et al. by analyzing the NGS *HLA-E* genotyping data of 2.5 million potential stem cell donors originating from 104 different populations (Sauter et al. 2021), and Lucas et al. by full-gene SMRT sequencing of *HLA-E* using 6227 DNA samples (Lucas et al. 2023). All studies clearly show that *HLA-E* is much more polymorphic than previously thought, but that the HLA-E*01:01 and *01:03 protein variants are by far the most common variants (~ 99%) with almost equal frequencies worldwide. This has already led to the suggestion that there is some balancing selection implying functional differences between these two protein variants (Olieslagers et al. 2017). A higher cell surface expression level for HLA-E*01:03 than *01:01 has been reported, due to its slightly higher peptide-binding affinity (Ulbrecht et al. 1999; Celik et al. 2016; Maier et al. 2000; Strong et al. 2003).

Although population studies on *HLA-E* typing are increasing, *HLA-E* variation has only been studied once in the Estonian population (Sauter et al. 2021), a relatively small Northern European country with around 1.3 million inhabitants. The Estonian population is known for its unique genetic profile, shaped by historical migrations, geographic isolation, and cultural influences, which have resulted in marked genetic structuring across the country, particularly between the South-East and other regions (Nelis et al. 2009; Pankratov et al. 2020). These historical and geographic factors have significantly influenced the general genetic diversity of Estonians. However, it remains unclear whether such structuring also affects *HLA-E* diversity within this population.

In this study, we aimed to comprehensively characterize the genetic variation of the *HLA-E* gene within the Estonian population by conducting full-length sequencing of samples from 143 individuals of Estonian origin, representing diverse regions of the country. By focusing on the Estonian population, we also explored potential regional differences in *HLA-E* variation and examined the association between *HLA-E* alleles and classical *HLA-A, -B,* and *-C* alleles. By focusing on this genetically distinctive population, our research contributes to the broader understanding of *HLA-E* diversity within populations and its implications for immune function.

## Materials and methods

For this study, we selected 143 DNA samples from the 1089 samples provided by the Estonian Biobank, which consists of 10,317 samples from individuals born in Estonia, collected in 2005 (Nelis et al. 2009). To ensure equal representation from each of the 15 Estonian counties, an equal number of individuals was selected per county, with comparable age distribution and gender between the counties. In total, the individuals age at the time of inclusion in the Biobank varied from 18 to 83, whereas 72 individuals were male and 71 female. The counties were grouped into different regions: South-East (Põlva, Tartu, Valga and Võru), North-East (Jõgeva, Ida-Viru and Lääne-Viru), and a group referred to as other than South-East (Harju, Hiiu, Ida-Viru, Jõgeva, Järva, Lääne, Lääne-Viru, Pärnu, Rapla, Saare and Viljandi) to compare regional differences within Estonia. The study was approved by the Ethics Review Committee on Human Research of the University of Tartu (216/T-10, 28.06.2012). Written informed consent for participation was obtained from all study subjects.

The complete *HLA-E* gene was amplified from 5’UTR to 3’UTR as previously described (Olieslagers et al. 2017). Amplicons were purified by ExoSAP-IT (Affymetrix, Santa Clara, California) according to the manufacturer’s protocol. Sanger sequencing was performed with both forward and reverse sequencing primers as described previously (Olieslagers et al. 2017).

Allele frequencies were obtained by direct calculation using the formula n/2N, where *n* is the number of observed alleles and *N* is the number of individuals analyzed. The allele frequencies (HLA-E*01:01:01G, *01:03:01G and *01:03:02G) of different regions were compared using the chi-squared test. The “G” suffix indicates that these alleles belong to the same G group, meaning they share identical amino acid sequences across the exons encoding the peptide-binding domains.

To determine associations between *HLA-E* alleles and the classical class I *HLA-A, -B* and *–C* alleles, low resolution typing of these loci was performed by Luminex SSO according to the manufacturer’s protocol using commercial kits (One Lambda, LABType SSO). For analysis of the data, PYPOP 0.7.0 was used (Lancaster et al. 2007). Of the 143 individuals, complete *HLA-A, -B*, and *-C* typing results were obtained for 128. These 128 samples were used for the association analysis. A standard chi-squared test, implemented in the PYPOP software package, was used to assess that data fitted into Hardy–Weinberg equilibrium.

## Results

### HLA-E allele and phenotype frequencies

The nucleotide polymorphism of *HLA-E* was studied in 143 Estonian individuals by full-length sequencing, covering the 5’UTR to the 3’UTR. Within this population, 4 alleles were identified that were unknown in the IPD-IMGT/HLA database at that time (HLA-E*01:01:01:51, *01:01:43, *01:01:01:53, and *01:01:01:54). The numbers and frequencies of *HLA-E* alleles found in this study are indicated in Table 1. The alleles HLA-E*01:01:01:01/*01:01:01:02, *01:06:01:01/*01:06:01:02, and *01:09:01:01/*01:09:01:02 were treated as a single group, as the amplicon sequence of the 5′ UTR region did not encompass positions − 181 to − 200, where the differences between these alleles are located. The *HLA-E* allelic distribution was in Hardy–Weinberg equilibrium with a *P*-value of 0.78 in the Estonian individuals. The allele HLA-E*01:01:01:01/02 (*f* = 0.441) was the most frequently observed, followed by *01:03:02:01 (*f* = 0.357).

**Table 1:** *HLA-E* allele numbers and frequencies in the 143 Estonian individuals

*HLA-E* allele	*n* =	Frequency
01:01:01:01/02	126	0.441
01:01:01:03	19	0.066
01:01:01:05	3	0.010
01:01:01:18	1	0.003
01:01:01:51	1	0.003
01:01:01:53	1	0.003
01:01:01:54	1	0.003
01:01:43	1	0.003
01:03:01:01	9	0.031
01:03:02:01	102	0.357
01:03:02:02	5	0.017
01:03:05:01	3	0.010
01:03:06	1	0.003
01:03:38	1	0.003
01:06:01:01/02	10	0.035
01:09:01:01/02	2	0.007

Within the study population four different HLA-E protein variants were found, HLA-E*01:01, *01:03, *01:06, and *01:09. Calculating the phenotype frequencies (Table 2) showed that 45% of the individuals are heterozygous for HLA-E*01:01 and *01:03. Comparing the protein sequences of these four HLA-E proteins reveals two distinct peptide-binding groove sequences: the groove of HLA-E*01:09 is identical to *01:01, with a difference located in the alpha 3 domain. Similarly, the groove of HLA-E*01:06 is identical to *01:03, with a difference located in the alpha 3 domain.

**Table 2:** HLA-E phenotype frequencies in the 143 Estonian individuals

HLA-E phenotypes		*n* =	Percentage
01:01	01:01	42	29%
01:01	01:03	65	45%
01:03	01:03	24	17%
01:01	01:06	4	3%
01:03	01:06	6	4%
01:03	01:09	2	1%

A previous study has shown that the Estonian population is genetically structured, with a strong distribution between South-East and North-East or South-East and other than South-East (Pankratov et al. 2020). Since our study population was spread over the 15 Estonian counties, we determined whether there were *HLA-E* allele frequency differences based on the high-resolution level (HLA*-*E*01:01:01G, *01:03:01G and *01:03:02G) between these regions. Within our study population, the chi-square values for the comparisons between the South-Eastern (*n* = 40) and North-Eastern counties (*n* = 27), and the South-Eastern and other than South-Eastern counties (*n* = 103), were 1.06 (*p* = 0.59) and 3.11 (*p* = 0.21), respectively, showing that no significant differences were observed between the allele frequencies of these regions (Table 3).

**Table 3:** *HLA-E* allele frequencies across different regions of Estonia

Region	*01:01:01G	*01:03:01G	*01:03:02G
South-East	0.50	0.08	0.43
North-East	0.61	0.11	0.28
Other than South-East	0.56	0.08	0.36

The present-day Estonian population has been shaped by various migration waves (Kivisild et al. 2021). To explore potential influences on *HLA-E* diversity, the allele frequencies observed in this study were compared with those reported for Estonians (*n* = 156) and neighboring populations, including Finns (*n* = 337), Russians (*n* = 14,896), Latvians (*n* = 301), Belarusians (*n* = 359), and Ukrainians (*n* = 2220), as described by Sauter et al. (2021). This study is based on DKMS registry donors in Germany, with ethnicity determined through self-assessment at recruitment. Additionally, comparisons were made with three Asian populations, Japanese (*n* = 245), Indonesian (*n* = 256), and Chinese (*n* = 980), as they are genetically distinct from European populations. Based on the high-resolution *HLA-E* alleles (*01:01:01G, *01:03:01G, and 01:03:02G), no major differences were observed between the Estonian samples from this study and the Estonian population reported by Sauter et al., nor between the Estonian and neighboring European populations (Table 4). As expected, the Estonian population showed clear differences compared to the Asian populations.

**Table 4:** *HLA-E* allele frequencies in studied Estonian individuals compared to the Estonian, neighboring and some Asian populations found by Sauter et al (2021)

*HLA-E* alleles	*01:01:01G	*01:03:01G	*01:03:02G	Comparison*
Estonia — this study (*n* = 143)	0.54	0.08	0.38	
Estonia — Sauter et al. (*n* = 156)	0.53	0.10	0.37	*p* = 0.6950
Finland — Sauter et al. (*n* = 337)	0.49	0.16	0.34	*p* = 0.0036
Russia — Sauter et al. (*n* = 14,896)	0.56	0.11	0.32	*p* = 0.0601
Latvia — Sauter et al. (*n* = 301)	0.55	0.08	0.37	*p* = 0.9572
Belarus — Sauter et al. (*n* = 359)	0.55	0.11	0.33	*p* = 0.1918
Ukraine — Sauter et al. (*n* = 2220)	0.57	0.13	0.30	*p* = 0.0034
Japanese — Sauter et al. (*n* = 245)	0.49	0.27	0.24	*p* < 0.0001
Indonesian — Sauter et al. (*n* = 256)	0.44	0.32	0.24	*p* < 0.0001
Chinese — Sauter et al. (*n* = 980)	0.42	0.28	0.30	*p* < 0.0001

*Chi^2^ calculated difference between Estonian population (this study) and the other populations

Given the close genetic relationship between the Estonian and Finnish populations, rooted in their shared historical and geographic ties (Kivisild et al. 2021), we further compared *HLA-E* variation between Estonians and Finns by analyzing SNP frequencies within the *HLA-E* gene among Estonian subjects from this study and Finnish individuals (*n* = 99) from the 1KG project (Table 5). This comparison utilized data from the publicly available third phase of the 1KG project (https://www.internationalgenome.org/data-portal/data-collection/phase-3), which provides whole-genome sequence data from 2504 individuals across 26 populations (Genomes Project et al. 2015). Overall, the SNP frequencies were similar across the two populations. However, there were some differences: at position 424, the T nucleotide (specific for HLA-E*01:03:02G) was more prevalent in the Estonian individuals compared to the Finnish (*f* = 0.378 vs 0.298), while at position 1857, the T nucleotide (specific for HLA-E*01:06) was less common in studied Estonian individuals compared to the Finnish population (*f* = 0.03 vs 0.14). When comparing the Estonian individuals and Finnish population, all evaluated SNPs present in the Finnish population were also found in the studied Estonian individuals, but not vice versa. Overall, no major differences were detected between the SNP frequencies of the populations examined.

**Table 5:** Comparison of SNP frequencies between studied Estonian individuals and the Finnish population

Location	5′UTR	E2	E2	E3	I3	E4	E4	E4	E4	E5	I5	I6	I6	3′UTR	3′UTR
gDNA position	− 104	409	424	756	1014	1625	1822	1857	1859	2076	2706	2924	2937	3224–3251	3500
major allele	A	G	C	A	T	G	A	C	C	C	C	C	G	A–#–C	T
minor allele	G	A	T	G	A	C	C	T	T	T	T	T	A	del	C
MAF Estonia	0.010	0.003	0.378	0.458	0.017	0.010	0.007	0.035	0.003	0.003	0.003	0.070	0.003	0.003	0.003
MAF Finnish	0	0	0.298	0.475	0.035	0	0	0.136	0	0	0	0.086	0	0	0

SNP frequencies from the Estonian individuals were calculated from our study, while SNP frequencies from the Finnish population were taken from the 1KG project. The minor allele frequency (MAF) is indicated

### Haplotypes/associations

In this study, we utilized the Pypop analysis tool to investigate the associations between *HLA-E* polymorphism R107G and other HLA class I alleles in the Estonian individuals. Although the R and G are present in almost equal numbers in our study, they are not equally distributed over the different *HLA-A* and *HLA-C* alleles (Table 6 (A and B)). HLA-A*01 was more frequently found with R than G (*R* = 12% vs *G* = 1%), HLA-A*03 was more frequently found with G than R (*R* = 2% vs *G* = 15%) and HLA-C*04 was more frequently found with G than R (*G* = 10% vs *R* = 0%). Also, the haplotypes A ~ B ~ C show a distinct association with either R or G, except for the haplotypes A*02 ~ B*27 ~ C*02 and A*02 ~ B*07 ~ C*07, which show an approximately equal distribution for R and G (Table 6 (C)). Furthermore, the HLA-B dimorphism in the leader peptide (− 21 methionine (M) or − 21 threonine (T)) showed no apparent pattern with R or G, suggesting no clear association.

**Table 6:** Association of *HLA-A* alleles, *HLA-C* alleles, HLA-A~B~C haplotypes, and HLA-B leader peptide dimorphism with amino acid position 107 of HLA-E

A							
HLA-A*	**R107**		**G107**				
	***n =***	**HF**	***n =***	**HF**			
01	30.6	0.119	1.4	0.006			
02	52.3	0.204	50.7	0.198			
03	6.1	0.024	37.9	0.148			
11	5.9	0.023	4.1	0.016			
23	N/A	0	2.0	0.008			
24	12.1	0.047	8.9	0.035			
25	9.0	0.035	N/A	0			
26	2.4	0.009	4.6	0.018			
30	3.7	0.015	1.3	0.005			
31	4.0	0.016	N/A	0			
32	8.0	0.031	N/A	0			
33	N/A	0	1.0	0.004			
68	3.9	0.015	6.1	0.024			
B							
HLA-C*	**R107**		**G107**				
	**n = **	**HF**	**n =**	**HF**			
01	6.0	0.023	9.0	0.035			
02	11.2	0.044	19.8	0.077			
03	14.9	0.058	24.1	0.094			
04	N/A	0	26.0	0.102			
05	11.0	0.043	2.0	0.008			
06	23.1	0.090	5.9	0.023			
07	48.0	0.188	26.0	0.101			
08	3.0	0.012	N/A	0			
12	8.4	0.033	1.6	0.006			
14	5.0	0.020	N/A	0			
15	6.3	0.025	2.7	0.011			
16	N/A	0	1.0	0.004			
17	1.0	0.004	N/A	0			
C							
HLA-A*	HLA-B*	HLA-C*	***HLA-B*** **leader allele**	**R107**		**G107**	
				***n =***	**HF**	***n =***	**HF**
01	08	07	M	17.9	0.070	1.1	0.004
03	07	07	T	1.4	0.006	12.7	0.050
02	27	02	T	6.2	0.024	6.7	0.026
03	35	04	M	N/A	0	11.8	0.046
02	07	07	T	3.8	0.015	6.4	0.025
02	44	05	T	8.8	0.034	1.2	0.005
02	13	06	M	9.7	0.038	N/A	0
02	40	03	T	N/A	0	8.5	0.033
02	15	03	T	N/A	0	5.9	0.023
03	15	03	T	N/A	0	6.0	0.023

Numbers (*n*) and haplotype frequencies (HF) for *HLA-A *alleles (pairwise LD=0.025, *p* < 0.000) (A), *HLA-C *alleles (pairwise LD=0.026, *p* < 0.000) (B), the 10 most frequent HLA-A~B~C haplotypes (C) and the HLA-B dimorphism in the leader peptide (C) associated with either R or G at amino acid position 107 of HLA-E

## Discussion

In this study, we performed full-length sequencing of the *HLA-E* gene in 143 Estonian individuals to identify all possible SNPs in both coding and non-coding regions, including the 5’ and 3’ UTRs, and identified a total of four novel alleles. The number of new alleles found in this small population is comparable to other studies that investigated full-length *HLA-E* polymorphism in specific populations (Castelli et al. 2015; Felicio et al. 2014; Olieslagers et al. 2017; Ramalho et al. 2017).

The genetic makeup of the Estonian population is shaped by a combination of factors, including historical migrations, genetic admixture, and geographic location, resulting in a complex blend that reflects both historical and contemporary influences (Pankratov et al. 2020; Kivisild et al. 2021). However, the results of our comparison of *HLA-E* genotype frequencies and individual *HLA-E* SNP frequencies with those of other populations indicate that these factors did not affect *HLA-E* variation. Our study utilized samples from individuals across different counties of Estonia as representatives of the whole Estonian population, allowing us to compare our findings with those of other populations. We found that *HLA-E* variation within Estonia was conserved across different regions and remained largely consistent when compared with their neighboring European populations. This suggests that the *HLA-E* gene serves a conserved function that is preserved across diverse genetic backgrounds, reflecting its crucial role in immune modulation.

The controversy between the low polymorphism of *HLA-E* opposite to the high polymorphism of the classical HLA class I genes, *HLA-A, -B*, and *–C*, has been the subject of speculation about the function and the evolutionary pathway of *HLA-E* (Geraghty et al. 1992; Grant et al. 2020). To get more insight in the latter one, we have evaluated the association of the classical class I HLA alleles with the dual polymorphism R107G of HLA-E. As can be deduced from Table 6, for some of the *HLA-A* or *–C* allele groups, there seems to be a preferential association with either R or G at amino acid position 107, like HLA-A*01 (R12% vs G1%) and HLA-A*03 (R2% vs G15%). In several of them, the association is rather exclusive, like HLA-A*25 (R4% vs G0%), *32 (R3% vs G0%), and HLA-C*04 (R0% vs G10%), although the small sample sizes could influence these observations. This is in agreement with the findings of Liu et al. (2012), who reported in a study on *HLA-E* in 4 different Chinese populations that several common *HLA-A* alleles displayed dichotomy of association with *HLA-E* alleles in all four populations. On the other hand, there are also *HLA-A* allele groups that can be present with both R and G, like HLA-A*02 and *24. In fact, Geraghty et al. already showed this phenomenon of restricted R107G associations with some of the *HLA-A* allele groups, like R with *01, but equal R/G distributions for other *HLA-A* alleles, like *02 (Geraghty et al. 1992). Although Geraghty found a restricted association of HLA-A*24 with R, Carlini et al. found equal distribution of HLA-A*24 with R and G, comparable to our results, in a study on linkage disequilibrium between *HLA-A* and non-classical HLA genes in 191 voluntary blood donors from south eastern France (Carlini et al. 2016). Since HLA-A*01 and *03 have identical peptide sequences from the leader peptide that could bind to HLA-E (VMAPRTLLL), this does not seem to be the trigger whether R or G is present on that haplotype. Taking the total A ~ B ~ C haplotype into account (see Table 6) an even more distinct association can be observed. Eight of the ten most frequent haplotypes show a clear preference for either R or G.

In conclusion, this study explored the polymorphism of the non-classical *HLA-E* gene within Estonian individuals, revealing several new SNPs and shedding light on its association with classical HLA class I genes. While the Estonian population’s genetic makeup reflects a blend of historical migrations and contemporary influences, *HLA-E* polymorphism appears to be conserved. The findings also highlighted preferential associations between specific classical *HLA-A* and *-C* allele groups and *HLA-E* polymorphism. These insights contribute to a clearer understanding of the conserved nature of *HLA-E* variation and its stable presence across diverse populations, irrespective of historical or regional genetic influences.

## Data Availability

The datasets generated during and/or analysed during the current study are available from the corresponding author on reasonable request.
